# Nanomaterials for Cardiac Tissue Engineering

**DOI:** 10.3390/molecules25215189

**Published:** 2020-11-07

**Authors:** Devang R. Amin, Eric Sink, Suguna P. Narayan, Mostafa Abdel-Hafiz, Luisa Mestroni, Brisa Peña

**Affiliations:** 1 Department of Internal Medicine, University of Colorado Anschutz Medical Center, Aurora, CO 80045, USA; devang.amin@cuanschutz.edu (D.R.A.); eric.sink@cuanschutz.edu (E.S.); 2 Department of Pathology, University of Colorado Anschutz Medical Center, Aurora, CO 80045, USA; suguna.narayan@cuanschutz.edu; 3 Department of Bioengineering, University of Colorado Denver, Anschutz Medical Campus, 12705 E. Montview Avenue, Suite 100, Aurora, CO 80045, USA; mostafa.abdel-hafiz@cuanschutz.edu; 4 Cardiovascular Institute, University of Colorado Anschutz Medical Campus, 12700 E. 19th Avenue, Aurora, CO 80045, USA; luisa.mestroni@cuanschutz.edu; 5 Consortium for Fibrosis Research & Translation, University of Colorado Anschutz Medical Campus, 12700 E. 19th Avenue, Aurora, CO 80045, USA

**Keywords:** cardiac tissue engineering, nanoparticles, carbon nanotubes, gold nanorods, cardiomyocyte regeneration

## Abstract

End stage heart failure is a major cause of death in the US. At present, organ transplant and left-ventricular assist devices remain the only viable treatments for these patients. Cardiac tissue engineering presents the possibility of a new option. Nanomaterials such as gold nanorods (AuNRs) and carbon nanotubes (CNTs) present unique properties that are beneficial for cardiac tissue engineering approaches. In particular, these nanomaterials can modulate electrical conductivity, hardness, and roughness of bulk materials to improve tissue functionality. Moreover, they can deliver bioactive cargo to affect cell phenotypes. This review covers recent advances in the use of nanomaterials for cardiac tissue engineering.

## 1. Introduction

Cardiovascular disease is a leading cause of morbidity and mortality across the United States and the world resulting in 800,000 and 17,000,000 annual deaths, respectively [1]. Atherosclerotic heart disease (AHD), in particular, is the primary driver of these deaths. AHD refers to the formation of atheromatous plaques within coronary vessels which are prone to rupture, resulting in myocardial infarctions (MI). In the last several decades, we have become increasingly adept at treating MIs through approaches such as percutaneous coronary intervention (PCI). Although this progress has improved the direct mortality from MI, the survivors of MI often progress toward heart failure (HF), which has continued to increase in prevalence. HF has become the most common diagnosis made in US adults over age 65, and approximately 6.2 million Americans above 20 years of age had heart failure from 2013–2016 [2]. The diagnosis of HF portends a downward cycle of declining quality of life, high healthcare costs, and shortened life expectancy [3,4,5]. HF commonly reaches a familiar endpoint, as over 300,000 US patients die annually from the disease [3,6].

There is a complex pathophysiology between myocardial infarction and heart failure. Although myocardial ischemia due to AHD or overt MI is the most common etiology of HF, it is important to note that there are other causes of HF. Ischemic insults result in gradual loss of functional myocardium and reductions in cardiac output. MI occurs when an atheromatous plaque ruptures within a coronary vessel, leading to occlusion of the vessel and loss of perfusion of the distal myocardium. If not intervened upon, the area of ischemic myocardium will infarct and progress through a series of well-defined healing stages [7]. This process initially includes acute inflammation and edema of the infarcted area, which then progresses to mononuclear inflammation, and, ultimately, collagen scar formation [8]. Scar within the myocardium represents areas of myocardial tissue death, translating to a decrease in the overall ability of the ventricles to generate cardiac output. Incremental declines in cardiac output induce pathologic neurohumoral activation, including upregulation of the renin-angiotensin-aldosterone signaling system, and also causes catecholamine excess [9,10]. In response to decreased cardiac contractility, these mechanisms attempt to maintain cardiac output by increasing baseline heart rate, increasing systemic vascular tone, and retaining sodium and water. However, elevated levels of circulating neurohumoral products initiate a process of ventricular chamber remodeling, which produces thinning of the ventricular walls and gross chamber enlargement [11,12]. Chamber enlargement begets a dangerous cycle of more neurohumoral activation and more remodeling. Medical therapy for HF centers around blocking this neurohumoral cascade and preventing chamber remodeling [13].

Advanced heart failure refers to patients with persistent HF symptoms that significantly inhibit their daily lives, despite maximal medical therapy. Of the 6 million HF patients in the US, approximately 200,000 meet the criteria for having advanced heart failure [14]. Patients with advanced heart failure despite medical therapy have limited options to improve cardiac output [15]. Myocardial tissue has very limited innate regenerative capacity, and no options are currently available for myocardial tissue regeneration. Current options for end organ intervention include either durable left ventricular assist device (LVAD) or heart transplant [16,17]. Both of these options require massive financial costs and major investment of time and effort by the patient and by medical care teams. Durable LVADs are implantable devices that sit within the cavity of the left ventricle. They use an inflow cannula and outflow cannula to move blood from the LV, through an external pump system, and then back into the systemic circulation [18]. While these devices have seen incremental improvements in the last several decades, they still entail major long-term risks and do not regenerate the heart [19,20]. Recent estimation of the rate of device thrombosis, gastrointestinal bleeding, and major stroke are 8 to 11%, 18 to 20%, and 13 to 29%, respectively [21]. Other adverse events include right ventricular failure and driveline infections.

Techniques in cardiac transplant have evolved such that transplant recipients now have an expected mean post-transplant life expectancy of 12–13 years [22]. Mortality is highest in the first several years after transplant, with one-year survival after adult transplant currently estimated to be 85%. Mortality is mostly related to primary organ failure, rejection, or infection related to immunosuppression [16]. Transplant entails lifelong immunosuppression, leaving the patient in a delicate balance between organ rejection and infection. Organ rejection accounts for as much as 10% of the deaths in transplant patients within three years after transplant [22]. Additionally, heart transplant as an intervention is limited by the number of available donors, a pool that cannot be readily increased [5].

The field of cardiac tissue engineering seeks to give patients with evidence of myocardial injury an additional therapy to potentially regenerate innate myocardial function. Nanomaterials, such as gold nanoparticles and carbon nanotubes, have unique electrical, mechanical, and thermal properties, which have positive therapeutic effects on electroactive cells, such as cardiomyocytes, which motivates the development of cardiac tissue engineering approaches. Moreover, they can serve as delivery systems for active biomolecules and/or drugs to further improve cardiac regeneration. This review covers recent advances in the use of nanomaterials for cardiac tissue engineering.

## 2. Nanoparticles for Medical Applications

Defined as materials with a characteristic dimension of 1–100 nm, nanoparticles have been increasingly investigated for medical applications, both as therapeutic and diagnostic tools [23,24]. Their size and high surface area-to-volume ratio make them particularly appealing for medical applications, enabling surface modification with high amounts of cargo or biomolecules and permitting their cellular uptake [25]. For example, chemotherapeutics or other drugs may be loaded onto nanoparticles by encapsulation or surface modification, and these nanoparticles may in turn be targeted to tumors, either passively by the enhanced permeability and retention effect or actively through the use of targeting moieties such as antibodies. Nanoparticles composed of a multitude of materials have been investigated for medical applications, including liposomes [26], gold [27,28], silver, iron oxide [29,30], carbon nanomaterials [31], organic polymers [32,33], metal-organic frameworks [34], and nucleic acids [35]. As of October 2017, 50 nanoparticles have been FDA-approved for medical applications, including liposome, polymer, and iron oxide nanoparticles [23].

Currently, most existing applications of nanoparticles in medicine rely upon intravenous injection of nanoparticles primarily for use in drug delivery or imaging. However, despite decades of work focused on delivery of nanoparticles to their target sites in vivo, the majority of intravenously-delivered nanoparticle doses do not actually reach target cellular niche. Chan and coworkers conducted a rigorous review of recent nanoparticle literature, finding that, on average, only 0.7% of nanoparticles targeted toward solid tumors reached their destination [36]. Nanoparticles with hydrodynamic diameter below 5.5 nm are cleared by the kidneys, and nanoparticles with larger dimensions are cleared by the mononuclear phagocyte system, primarily in the liver and spleen. Several investigations have been focused on the clearance mechanisms of nanoparticles by the body, which hinder their delivery to sites of interest. For example, Tsoi et al. conducted an in vivo and computational mechanistic study evaluating clearance of hard quantum dot and gold nanoparticles, showing that these nanoparticles are efficiently cleared primarily by Kupffer cells (specialized macrophages) in the liver and by splenic macrophages [37]. Liver clearance is highly efficient, due to the relative movement of nanoparticles in the diffusion regime, rather than advection regime in liver sinusoids, where blood flow drops 1000-fold from that of incoming blood vessels. This efficient clearance complicates systemic delivery of nanoparticle-based therapeutics, promoting interest in systems of localized delivery, which evade the problem of rapid clearance.

The incorporation of nanoparticles into tissue engineering has been developing more recently [38,39]. Within cardiac tissue engineering, nanomaterials may have particularly high utility. Cardiac tissue engineering focuses on supporting injured cardiac tissue, sometimes by inducing proliferation of cells with regenerative potential, which include embryonic stem cells, mesenchymal stem cells, or induced pluripotent stem cells [40,41]. Many of these efforts focus on development of polymer patches or scaffolds that can support tissue regeneration or repair. Biomaterials that have been tested for cardiac tissue engineering include 3D-printed cardiac patches, electrospun polymers, and hydrogels [40,41]. Injectable hydrogels have also been formulated to make clinical use more convenient, permitting facile delivery to injured myocardium without the need for invasive approaches [42]. These materials may subsequently be modified with bioactive molecules, such as peptides, microRNA, or growth factors. Although these biomolecules may be degraded under physiological conditions, nanoparticles can help to stabilize them. Nanoparticles have been found to be useful in such efforts by stimulating neighboring cells and by serving as platforms for modification with bioactive molecules [38]. This review highlights recent research into the effects of nanoparticles on the growth of cardiac tissue, both from an empiric and mechanistic perspective. Research demonstrates that nanoparticles are able to affect cardiac tissue regeneration or repair by their inherent mechanical and electrical properties, as well as through their ability to incorporate bioactive molecules that participate in natural biochemical signaling.

In the upcoming sections of this review paper, we discuss the specific studies evaluating the incorporation of nanomaterials into cardiac tissue engineering approaches. We place special emphasis on carbon nanostructures and noble metal nanoparticles, which have been studied most extensively. In particular, carbon nanotubes and gold nanorods have spurred the most research efforts, and are discussed in the most detail.

## 3. Carbon Nanomaterials

Carbon nanomaterials are biomaterials composed predominantly of carbon atoms with one dimension less than 100 nm. These nanomaterials fall into three categories: graphene, carbon nanotubes (CNTs), and fullerene, all of which have been discovered within the last four decades [31]. Graphene is a 2D sheet of carbon atoms, and CNTs consist of sheets of carbon atoms rolled into cylindrical form. Depending on the number of carbon sheets wrapped around their axis, CNTs may either be single-walled (SWCNTs) or multi-walled (MWCNTs) structures. SWCNTs contain one layer of carbon atoms while MWCNTs contain multiple layers of carbon atoms. Fullerene is a spherical assembly of carbon atoms. These materials are electrically conductive (or semi-conductive), mechanically stiff and strong, and can be modified with hydrophobic substrates, making them useful for applications across multiple industries.

As discussed in the excellent review paper by Hong et al., these interesting properties of carbon nanomaterials are due to their unique chemical structures [31]. Specifically, these materials consist of sp^2^ hybridized carbon atoms, in contrast with bulk carbon materials such as diamond and graphite, which contain sp^3^ hybridized carbon atoms [31]. The hybridization of carbon atoms in sp^2^ geometry leaves open one p orbital per carbon atom, perpendicular with the plane of carbon atoms in these structures. These p orbitals can combine to form planar π molecular orbitals, which enable the sharing of electrons between many carbon atoms, resulting in the electrical conductivity (or semi-conductivity) of CNTs and graphene nanostructures. Additionally, these π orbitals permit the modification of carbon nanomaterials via π-π stacking with hydrophobic cargo. These materials also have high mechanical strength and stiffness. Furthermore, CNTs and graphene are anisotropic nanostructures that promote alignment of electrical cells, such as cardiomyocytes, preferentially in one direction. This increase in contractility of cells along the long axis mimics cardiac tissue. The conductivity, anisotropy, and mechanical strength of carbon nanostructures have been explored for therapeutic approaches in cardiomyocytes.

**Figure 1:**
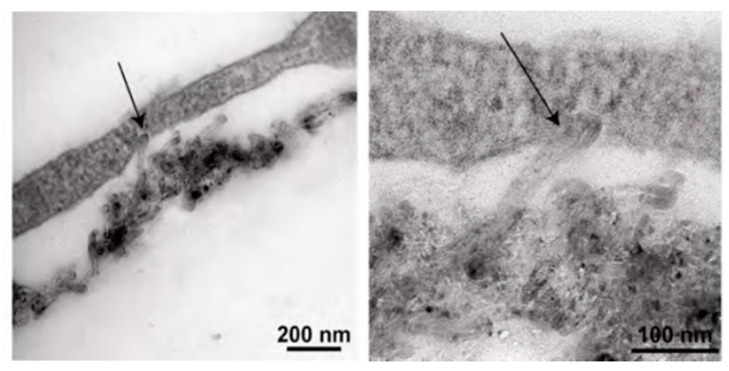
Carbon nanotube (CNT) interaction with cardiac myocyte membranes (arrows). The images show a tight interaction between the CNT and the cardiomyocyte cell membrane. Reprinted with permission from [43] Copyright 2012 American Chemical Society.

CNTs induce cardiomyocytes to function in a way that improves cardiomyocyte activity for cardiac tissue applications. Multiple studies have been conducted via electron microscopy and biochemical techniques in order to evaluate cardiomyocyte phenotype changes, due to the use of CNTs. Our laboratory’s work by Martinelli et al. in 2012 was the first to demonstrate the therapeutic effects of CNTs on electrophysiological behavior of cardiomyocytes [43]. Our laboratory reported that CNTs were particularly helpful to promote neonatal rat ventricular cardiomyocyte (NRVM) proliferation, maturation, and improved electrical behavior [43,44,45]. By conducting transmission electron microscopy (TEM), Martinelli et al. found that MWCNTs formed tight contacts with NRVM cell membranes, as shown in Figure 1 [43]. Over the course of three days, when compared to NRVMs grown on gelatin, NRVMs grown on CNTs had greater overall proliferation, more negative resting membrane potentials, and greater ability to spontaneously fire action potentials. Upon probing the biochemical mechanisms of this behavior, it was found that NRVMs grown on CNTs had greater α-MHC (myosin heavy chain) expression and lesser β-MHC expression, corresponding to an adult cardiomyocyte phenotype [45]. Sarcoplasmic reticulum Ca^2+^ ATPase (SERCA) and connexin 43 (Cx43), associated with gap junctions, were both also increased in the NRVMs grown on CNTs. Sun et al. identified additional components of signaling involved in cell-CNT interactions, finding that CNTs embedded in a gelatin methacrylate anhydride gel induce β1-integrin-mediated FAK (focal adhesion kinase) and RhoA (a cytosolic GTPase protein) upregulation in electrical and mechanical junction proteins [46]. These signaling pathways promote intercalated disc formation. These biological interactions between carbon nanomaterials and cardiomyocytes continue to be actively explored.

Anisotropy has been harnessed in the field of cardiac tissue engineering as a whole, in order to improve biological outcomes, and CNTs provide an opportunity to impart anisotropy to materials. For example, 3D printed materials have been built with anisotropic properties that have guided the formation of endothelialized myocardium in a “heart on a chip,” which is capable of contracting [47]. In this approach, an ink of gelatin methacryloyl (GelMA), alginate, and cross-linker (Irgacure) was utilized to create a microfibrous scaffold for tissue growth. Ramón-Azcón et al. used the idea of anisotropy by dielectrophoretically aligning CNTs in GelMA in order to fabricate contractile cardiac tissues from C2C12 myoblasts [48]. By aligning CNTs in one direction using an electrical field, Ramón-Azcón et al. also created a material with increased conductivity that promoted expression of α-actinin and myosin heavy chain proteins in C2C12 myoblasts. In turn, the myofibers grown from these myoblasts on CNT-containing hydrogels had greater contractility than those grown on CNT-free hydrogels, as assessed by myotube displacement in vitro. In this manner, the combination of intrinsic CNT anisotropy can be paired with materials synthesis techniques like dielectrophoretic alignment to further take advantage of this property for tissue engineering.

The use of electrical and mechanical properties of CNTs to influence stem cell differentiation and cardiomyocyte phenotypes in hydrogels has also been investigated. Ahadian et al. used a gelatin methacryloyl gel with aligned CNTs to differentiate embryoid bodies into cardiomyocytes. They achieved this by controlling the frequency, amplitude, and duration of an electrical stimulation through the gel [49]. This approach relies on dielectrophoretic alignment of CNTs in the gel to increase the gel conductivity. Subsequently, the authors conducted a study in which embryoid bodies were directly exposed to CNTs in cell culture medium, finding that this exposure to CNTs was also capable of inducing embryoid body differentiation into cardiomyocytes [50]. The authors observed that integrins involved in the mechanotransduction pathway were upregulated in the embryoid bodies exposed to CNTs, suggesting a possible mechanism for this phenomenon. Using atomic force microscopy, the investigators found that the Young’s moduli of embryoid bodies with CNTs are higher (35.2 ± 5.6 kPa), in comparison with embryoid bodies alone (20.9 ± 6.5 kPa). In another study, Ahadian et al. were able to incorporate CNT into poly(octamethylene maleate (anhydride) 1,2,4-butanetricarboxylate) gels [51]. They found that by using a 0.5% concentration of CNTs they could achieve greater electrical conductivity than when using 0.1% and 0% CNTs. NRVM cells seeded onto the 0.5% CNT hydrogel showed more synchronized beating and a greater excitation threshold, suggesting more mature tissue [51]. PLGA-carbon nanofiber composites have also been found to have increased primary human cardiomyocyte proliferation, hypothetically due to electrical conductivity [52]. This proliferation was assessed in vitro over 5 days. The same trend held for rat neuroblastoma neurons, which are electrically active, like cardiomyocytes. These studies all show the utility of CNTs for cardiac tissue engineering, highlighting the importance of mechanotransduction, electrical conductivity, and mechanical properties in directing the differentiation and development of cells.

Mechanical properties of CNTs have been investigated rigorously as cues for cardiomyocyte phenotype in other work. Yu et al. were able to show improvement in NRVM cell function by incorporating CNTs into type I collagen hydrogels [53]. In the study the authors showed that collagen hydrogels with 2 wt% CNTs had Young’s moduli of 1.85 ± 0.44 kPa, closely matching those of neonatal rat heart tissue (4.0–11.4 kPa). These gels also had reduced impedance versus those without CNTs. When NRVMs were seeded onto collagen hydrogels with 2 wt% CNTs, a larger percentage of the scaffold area was covered with rhythmically contracting NRVMs compared to hydrogels with lower or higher CNT concentrations, suggesting that the mechanical and electrical properties of CNTs promote NRVM functionality. In another effort to modulate mechanical properties of hydrogels, Ho et al. were able to incorporate CNTs into polycaprolactone hydrogels by 3D printing scaffolds, formulating scaffolds with higher maximum load, elastic modulus, and hardness than those without CNTs [54]. These scaffolds were also shown not to be harmful to H9C2 cells [54]. These studies show that mechanical properties of scaffolds may be modulated by adjusting CNT content, which allows for the tuning of biological response.

The influence of conductivity on atrial versus ventricular cardiomyocyte phenotype has also been investigated. Lee et al. performed a mechanistic study using GelMA scaffolds containing various CNTs, graphene oxide (GO), and reduced graphene oxide to evaluate the effects of conductivity on NRVM cell phenotypes on these scaffolds [55]. This group found that the CNT-containing scaffolds, which were most conductive, induced a ventricular-like phenotype, while the GO-containing scaffolds, which were less conductive, induced an atrial-like phenotype. Reduced GO scaffolds induced a mixed phenotype.

The method of preparing CNT-containing materials also plays a critical role in outcome. For example, Liu et al. created poly(ethylene glycol)-poly(D,L-lactide) copolymer (PELA) fiber scaffolds using electrospinning, finding that the method used to incorporate the CNTs into the polymer had an effect on NRVM cell viability and function. If the CNTs were incorporated into the scaffolds coaxially within inner portions of the PELA fibers, the cells showed greater elongation and increased α-actinin and troponin I expression, versus materials with CNTs blended throughout the PELA, even though the PELA fibers with CNTs blended into them had greater conductivity and Young’s moduli. This study also found that cardiomyocytes on a coaxial scaffold with 5% CNT showed the best performance in terms of cell maturation, and this scaffold had lower toxicity [56].

Electrospinning has been explored further by other investigators, who have taken advantage of the anisotropy of electrospun materials. Kharaziha et al. were able to incorporate CNTs into an aligned poly(glycerol sebacate):gelatin gel. They found that this scaffold had greater electrical conductivity and toughness compared to scaffolds without CNT. NRVM cells grown on this scaffold had more synchronous beating behavior and maintained cell viability [57]. Wu et al. used electrospinning to make networks of “nanoyarns” composed of PCL, silk fibroin, and CNTs, which were then coated in a gelatin methacrylate gel [58]. When seeded with NRVMs and endothelial cells in vitro, these hydrogels containing nanoyarn networks supported the formation of endothelialized myocardium. Mombini et al. electrospun chitosan/polyvinyl alcohol mesh with embedded CNTs [59]. They found that a 1% CNT concentration was best for promoting differentiation of mesenchymal stem cells into cardiomyocytes. Using this gel and an applied current, they showed that there was a significant increase in the differentiation of mesenchymal stem cells into cardiac cells [59].

Several other investigators have constructed CNT-containing hydrogels that have achieved favorable outcomes in vitro and in vivo. Pok et al. showed that a chitosan hydrogel with CNT can support beating NRVM cells at almost natural beating rates. The cells on the hydrogel also showed synchronous beating due to the improved conductivity of the gel [60]. In another study, Shin et al. showed that NRVMs seeded on a gelatin methacrylate gel with CNT had a lower excitation threshold and more synchronous beating [61]. In an animal study Zhou et al. used a CNT/gelatin matrix to show improved cardiac function post infarct [62]. In this study, LAD infarct rat models were used, and fusion of the CNT/gelatin material was observed with the infarcted myocardium. Left ventricular fractional shortening and ejection fraction both improved after applying CNT/gelatin materials versus sham control, and they improved more greatly when compared with gelatin that did not contain CNTs.

Growth factors can be delivered by modification of hydrogels containing carbon nanomaterials in order to optimize their properties. Paul et al. constructed GelMA hydrogels containing graphene oxide as a means of delivering plasmid DNA of VEGF to transfect adjacent endothelium and stimulate angiogenesis in peri-infarct regions of rat models [63]. This strategy was effective in induction of tissue revascularization and improving contractile performance, as assessed via echocardiogram.

Fullerene-derived carbon nanomaterials have also been studied for cardiac tissue engineering. The fullerene derivative fullerenol has been incorporated into injectable alginate hydrogels to improve the survival and proliferation of brown adipose derived stem cells and promotes their differentiation into cardiomyocytes [64]. Hao et al. proposed that this phenomenon occurred through the materials’ antioxidant activity, which reduced oxidative stress damage that is ordinarily caused by ROS, following MI. These materials were shown to reduce infarct size, increase wall thickness, and increase left ventricular ejection fraction in an MI rat model.

Stem cell encapsulation within CNT-containing gels has been explored to improve integration with heart tissue. Li et al. modified a poly (N-isopropylacrylamide) with CNT [65]. The gel was used to encapsulate brown adipose derived stem cells. This was tested in a myocardial infarction rat model, and was shown to improve the implantation of the cells, as well as increase their proliferation [65]. Additionally, left ventricular ejection fraction and fractional shortening were both higher in animal models treated with CNT-containing hydrogels versus those treated with CNT-free hydrogels. These trends were correlated with decreased infarction area and increased wall thickness in animals treated with CNT-containing hydrogels at 4 weeks post-implantation.

**Figure 2:**
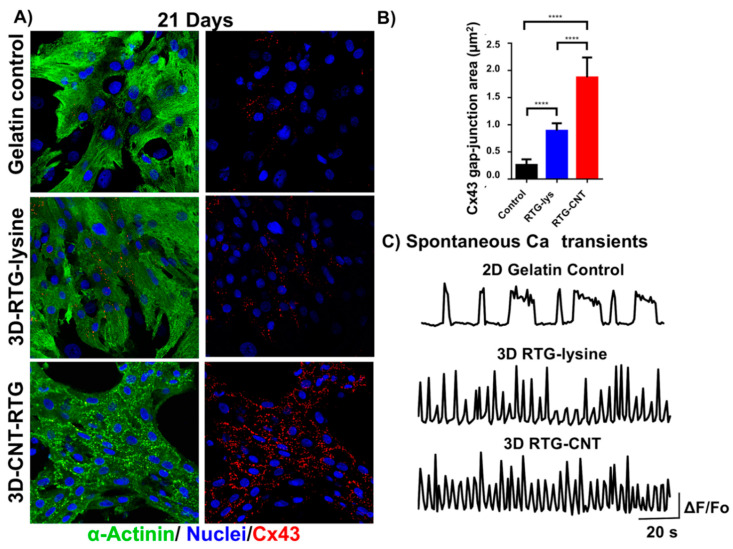
Intercellular communication of neonatal rat cardiomyocytes (NRVMs) growing in different substrates after 21 days of culture. (**A**) Fluorescence images of connexin 43 (red dots), sarcomeric a-actinin (green) and DAPI (blue) staining of NRVMs: top-row panels NRVMs cultured on 2D gelatin control; middle-row panels NRVMs cultured in 3D reversible thermal gel (RTG)-lysine; bottom-row panels NRVMs cultured in 3D RTG-CNT. (**B**) Quantification of Cx43 gap junction area: Significant differences on Cx43 gap-junction were observed between the gelatin control groups and the RTG systems. RTG-lysine “vs.” gelatin control **** *p* value: <0.0001, *n* = 8; RTG-CNT “vs.” gelatin control **** *p* value: <0.0001, *n* = 8; RTG-CNT “vs.” RTG-lysine **** *p* value: <0.0001, *n* = 5. Data are presented as mean ± S.D. (*n* = 5). (ANOVA-Bonferroni’s test). Data are presented as mean ± S.D. (*n* = 5). (**C**) Spontaneous calcium transients of NRVMs growing on 2D gelatin control and in 3D RTG systems (*n* = 5). Reprinted with permission from [66] Copyright 2017 American Chemical Society.

The route of administration of CNT-containing materials has been considered in some studies, as non-invasive methods may be preferable for future clinical applications. Our research group has also developed a unique injectable formulation of CNTs chemically incorporated into a reversible thermal gel (RTG) that is liquid at room temperature and gels at body temperature [66]. This work on 3D culture of NRVM cells was driven by the finding that CNTs in 3D scaffolds increased synaptic activity of cardiomyocytes versus 2D gelatin coated plates and 3D scaffolds without CNTs [67]. In comparison with 2D gelatin controls and the 3D scaffolds without CNTs, the culturing of NRVMs in the 3D CNT-RTG hydrogel resulted in greater proliferation of cardiomyocytes relative to fibroblasts. The cardiomyocyte gap junctional area, as assessed by a Cx43 assay, was also increased in the cells cultured in the 3D CNT-RTGs versus cells cultured in both 2D gelatin and 3D RTGs without CNTs, as shown in Figure 2A,B. In addition, cardiomyocytes grown on the 3D CNT-RTG presented a more homogeneous calcium transients when compared with NRVMs cultured in 2D-gelatin controls and NRVM cultured in the RTG without CNTs (Figure 2C). These 3D CNT-RTGs improve cardiomyocyte function, while allowing for non-invasive administration.

Unique from the aforementioned studies, CNTs have also been evaluated in vivo using models other than myocardial infarction models. In one recent study, McCauley et al. ablated the atrioventricular nodes of sheep models, and CNT fibers were sutured across the scars created by this process [68]. Although this study with CNT fibers did not involve cardiac tissue engineering per se, it did demonstrate that the electrical conductivity of CNTs could be quite helpful in damaged cardiac tissue. These CNT fibers restored conduction through the scarred area in sheep. In the same study, McCauley et al. sewed CNT fibers from the lateral right atrium to the lateral right ventricle in several rat models. The authors found that there was pre-excitation of the right ventricle in the acute setting, but this pre-excitation stopped after 4 weeks. The conclusions of this study interestingly demonstrate for the first time that CNT fibers can reconstitute electrical conduction in the heart.

It should be noted that some studies involving CNTs showed that there is significant toxicity associated with their use in vivo, as reviewed by Liu et al. [69]. Initial studies reported pulmonary toxicity caused by inflammatory and fibrotic reactions to the presence of CNTs. CNTs can enter cell lysosomes, resulting in oxidative stress and inflammation, which mediate toxic effects [70,71]. These studies may not have relevance to most biological applications, which do not involve inhalation of CNTs. Later studies have shown that surface chemistry is the driving force behind CNT-associated toxicity. CNTs functionalized with nontoxic surface molecules were shown to have no significant effects on serum chemistry and organ histology of mice that were intravenously injected with PEGylated-CNTs over three months [72]. In applications such as the above, which involve the implantation of CNTs embedded in scaffolds, the toxic effects may be reduced since CNTs are localized and stabilized in the region of interest. The aforementioned in vivo studies for cardiac tissue regeneration using CNTs in this section demonstrated therapeutic effects, which demonstrates that at the very least, the therapeutic effects for such applications outweigh the toxic effects over the time-scales of these experiments.

Overall, carbon nanomaterials have been investigated in great depth for cardiac tissue engineering, although the mechanism by which tissue engineering is achieved remains unclear and is perhaps multifactorial. Mechanotransduction, conductivity, anisotropy, and mechanical properties, such as Young’s modulus, are among the possible factors at play. Regardless, outcomes for cardiac tissue repair for carbon nanomaterial-containing scaffolds have been favorable in both in vitro and in vivo studies.

## 4. Noble Metal Nanoparticles

Gold nanoparticles (AuNPs), gold nanorods (AuNRs), and silver nanoparticles have been studied extensively for biomedical applications [24,27,28]. Via redox reactions from cationic gold salts, gold nanoparticles can be designed in a broad range of shapes, including spherical nanoparticles and cylindrical nanorods. These materials are notably resistant to oxidation and are relatively inert, which make them attractive options for biological applications. Despite this inertness, gold nanostructures can be modified by utilizing the strong gold-thiolate bond with either thiols or disulfides [28].

Mixing AuNRs into hydrogels enhances cardiomyocyte differentiation into functional tissue. One method of incorporating AuNRs into hydrogels for tissue engineering is via mixing of AuNRs with UV-crosslinkable of gelatin-methacrylate (GelMA) prepolymers [73]. Compared with AuNR-free GelMA control hydrogels, addition of AuNRs into GelMA at low concentrations (1.5 mg/mL) decreased impedance, increased Young’s modulus up to ~1.3 kPa from ~450 Pa for pure GelMA, and decreased hydrogel swelling ratio and pore diameter. When exposed to NRVMs in vitro, up to 50–60% of the area was covered by cardiomyocytes within 1 day in GelMA containing 1.5 mg/mL AuNRs versus <20% for GelMA without AuNRs, and these cells were more viable. Furthermore, NRVMs were more organized in GelMA with AuNRs as assessed by morphology and staining for F-actin, α-actinin, and connexin 43. NRVMs grown on AuNR-containing GelMA also exhibited increased synchrony of spontaneous beating at faster rates, with those hydrogels containing 1.5 mg/mL AuNR reaching 102 ± 72 beats per minute (bpm) at 7 days, versus 33 ± 9 bpm for control. In subsequent work, Navaei et al. incorporated parallel grooves 50 µm in width and depth into AuNR-containing GelMA using a lithographically-patterned PDMS mold [74]. Based on images included in the publication, the incorporation of micro-scale grooves qualitatively appears to induce more aligned sarcomeres of NRVMs in vitro than the earlier study. As in their prior study, GelMA containing AuNRs had increased cell density, and they also had significantly increased connexin 43 and α -actinin coverage area. NRVMs grown on these materials were found to demonstrate spontaneous beating over days 4 to 7 of culture.

Navaei et al. have since performed a fascinating study to evaluate what property of the AuNRs in GelMA hydrogels is most beneficial for biological outcomes [75]. In this work, the authors found that the incorporation of both AuNRs and silicon nanoparticles into GelMA both resulted in similar effects on NRVM growth and phenotype, as assessed by overall cell area and by staining for connexin 43, cardiac troponin I, and α-actinin. At concentrations used, silicon nanoparticles did not increase the bulk Young’s modulus or conductivity versus GelMA control. This interesting result suggests that the mere incorporation of nanoparticles may be responsible for effects on cardiomyocyte growth, independent of any influence on conductivity or bulk material stiffness.

AuNRs have also been incorporated into collagen scaffolds [76]. These collagen scaffolds were relatively stiff (elastic modulus 6.56 kPa ± 0.07), and the incorporation of AuNRs into these matrices did not significantly increase the bulk elastic modulus (7.25 kPa ± 0.08). Based on atomic force microscopy, however, AuNRs did increase stiffness at the nano-scale. The presence of these AuNRs enhanced intercalated disc formation, as evaluated by staining for intercalated disc proteins (connexin 43, N-cadherin, plakophilin 2, and plakoglobin) in NRVMs. The authors also showed that intercalated disc formation was driven by the β1 integrin-mediated ILK/p-AKT/GATA4 signaling pathway, as anti-β1-integrin antibody blocked intercalated disc formation, and downstream targets of β1-integrin (p-FAK, p-Src, ILK) were phosphorylated when exposed to AuNR-containing collagen.

Spherical AuNPs have also been studied for cardiac tissue engineering. Baei et al. evaluated the effect of AuNPs added to chitosan thermosensitive hydrogels, which were seeded with mesenchymal stem cells (MSCs) [77]. AuNPs increased conductivity of chitosan hydrogels, and also increased gelation time in a concentration-dependent manner. There was no difference in proliferation of MSCs over 14 days of growth, but there was a significant increase in the expression of cardiac-specific markers Nkx-2.5 and α-MHC in cells grown on chitosan gels containing 1 wt% AuNPs versus those without AuNPs.

Nair et al. integrated AuNPs into a porcine gall bladder-derived extracellular matrix to assess effect on H9C2 cardiomyoblast growth in vitro [78]. These researchers found no difference in quantitative cell viability on the extracellular matrix with and without AuNPs, with >70% H9C2 cell viability at 3 days across all substrates.

Modification of Au and AgNPs has also been performed, in order to influence tissue scaffold architecture. For example, Alarcon and coworkers experimented with the incorporation of peptide-coated AuNPs and AgNPs into injectable collagen-containing hydrogels to evaluate their effect on NRVM growth and macrophage polarization [79]. The authors found that the viscosity of the materials containing peptide-coated AuNPs were approximately four times greater than other gels, and electrical conductivity was increased by incorporation of either set of NP into the scaffolds. Scaffolds containing AgNPs and AuNPs favored M2 polarization of macrophages in vitro. Interestingly, these scaffolds did not cause greater connexin 43 expression in NRVMs in the absence of electrical stimulation, although this trend was observed when electrical stimulation was applied for 24h in comparison to NRVMs grown on NP-free collagen scaffolds. The same research group previously reported that AgNPs could be electrospun with collagen into a fibrous material that similarly increased connexin-43 expression in NRVMs under electrically stimulation, but this material did not activate macrophages [80].

**Figure 3:**
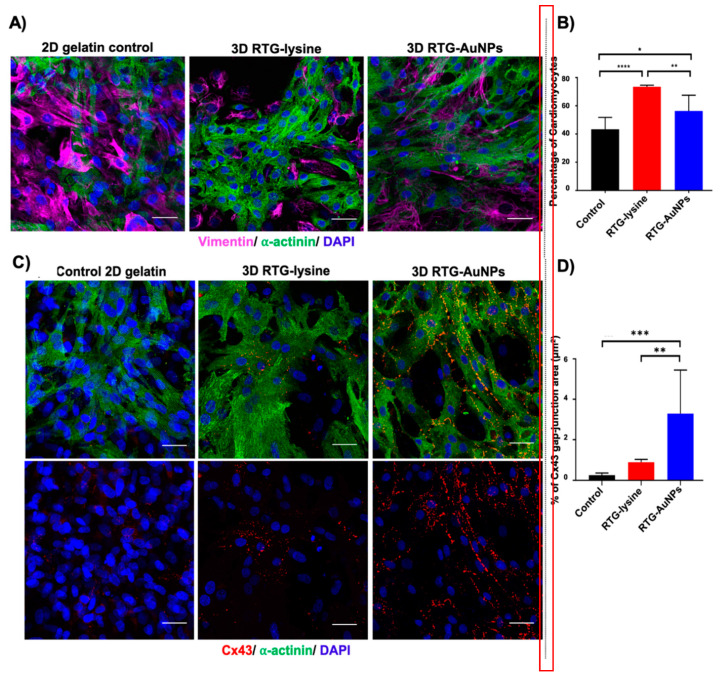
Upper panel: Immunocytochemistry labeling of NRVMs and cardiac fibroblasts (CFs) cultured in 2D and 3D systems for 21 days. (**A**) Antibody staining against α-actinin (green) and vimentin (pink) label NRVMs and CFs, respectively, with nuclei labeled using DAPI (blue). (**B**) Quantification of immunocytochemistry staining against α-actinin indicates percentage of cells likely to be NRVMs, showing both 3D systems to contain a greater percentage of NRVMs than the 2D gelatin control. Scale bar 40 µm. *p* values: * < 0.023, ** < 0.0017 and **** < 0.0001. Data are presented as mean ± S.D. Lower panel: Immunocytochemistry labeling of gap junctions in NRVMs cultured in 2D and 3D systems for 21 days. (**C**) Antibody staining against connexin 43 (Cx43) (red) and α-actinin (green), with nuclei labeled using DAPI (blue). (**D**) Quantification of immunocytochemistry staining against Cx43 to indicate surface area of NRVMs positive for this gap junction protein, showing the RTG-AuNPs system to contain the largest Cx43-positive area. Scale bar: 40 μm. *p* values: ** < 0.0021 and *** < 0.0002. Data are presented as mean ± S.D. Reprinted with permission from [81] Copyright 2019 American Chemical Society.

Our group has recently investigated reverse thermal gels (RTGs) containing AuNPs for in vitro growth of NRVM cells [81]. RTGs are composed of poly(serinol hexamethylene urea)-co-PNIPAAm and have the property of being injectable due to a sol-gel transition temperature between 25 °C and 37 °C. The chemical incorporation of AuNPs into the RTG backbone decreased their electrical resistances (333.7 kΩ ± 50.5 for RTG only controls versus 140.1 kΩ ± 34.9 for RTG-AuNP gels). In co-cultures of NRVMs with cardiac fibroblasts on these materials, it was found that, when compared with 2D gelatin controls, both RTGs and RTG-AuNP gels supported growth of an increased percentage of cells with cardiomyocyte phenotype, as assessed by staining for α-actinin (cardiomyocyte-specific) and vimentin (cardiac fibroblast specific) (Figure 3A,B). Furthermore, when NRVMs were cultured on these RTG-AuNP gels, there was an increase in gap junction area as assessed by connexin 43 staining versus RTG-only gels at 21 days of growth (Figure 3C,D). These results demonstrate that AuNPs provide a cue within RTGs that promotes gap junction formation between cardiomyocytes.

## 5. Polymer Nanoparticles

Polymer nanoparticles have been investigated as well, though in significantly less depth than the above carbon nanomaterials or noble metal nanomaterials. One advantage of polymer nanoparticles is that they can be readily modified with drugs and biomolecules by a broad range of chemical techniques. These systems also tend to be more degradable, theoretically resulting in reduced toxicity. However, they do not have the same level of conductivity or hardness as the previously discussed nanomaterials.

There has been a particular emphasis on the use of nanomaterials that use mussel-inspired dopamine or polydopamine chemistry. Developed by Messersmith et al., these chemical strategies draw inspiration from the use of the marine mussel *Mytulis edulis*. This sea creature must withstand high forces to adhere to rocks, relying on adhesive foot proteins that are composed of high quantities of the catechol-containing amino acid 3,4-dihydroxy-l-phenylalanine (DOPA) [82,83]. The catechol (3,4-dihydroxyphenyl) group interacts strongly with TiO_2_ and amine-terminated silica surfaces [84]. Based upon the adhesive importance of the catechol, PDA was synthesized by the incubation of the catecholamine dopamine in aqueous conditions at alkaline pH [85]. The versatile chemistry of PDA can be utilized for facile modification with thiols and amines. Thiols may bind to the catechol by Michael addition onto oxidized quinones present in the PDA [86]. Amines may also bind via Michael addition, or they can bind via Schiff base reactions, enabling bioconjugation with proteins [87]. Metals can also be deposited on the PDA by reduction of metal salts. This chemical versatility has been utilized to conjugate many different molecules onto PDA and related catecholamine materials over the years, such as DNA [88], PEG [85], hydroxyapatite for biomineralization [89], and RGD peptides for cell attachment [90]. PDA has also been formulated into nanoparticles, retaining these properties for facile modification while also demonstrating antioxidant activity [91,92,93,94,95].

Wang et al. designed a self-assembled nanoparticle composed of a bioactive drug (tanshinone IIA), coated this nanoparticle with PDA, and attached it to an ROS-polymer hydrogel via PDA-thiol bonds [96]. The resulting hydrogel material was injected into the cardiac tissue of myocardial infarction rat models. When implanted, the drug-loaded nanoparticles degraded in vivo, resulting in enhanced left ventricular ejection fraction and fractional shortening.

Conductive polypyrrole nanoparticles were linked using mussel-inspired chemistry to generate a cryogel with GelMA [97]. When implanted as a patch on infarcted myocardium in myocardial infarction rat models, this material increased left ventricular ejection fraction by about 50% and reduced infarct size by 42.6%. A similar scaffold of electrospun GelMA/polycaprolactone with GelMA-polypyrrole nanoparticles was made with dopamine-MBA cross-linker, resulting in a 50% decrease in infarct area and 20% increase in left ventricular shortening fraction percent in myocardial infarction rat models [98].

In another approach, carboxymethylcellulose nanoparticles were used by Kim et al. to induce differentiation of mature normal human dermal fibroblasts into induced cardiomyocyte cells by delivery of the drug 5-AZA to stop proliferation and plasmid DNA (GATA4, MEF2C, and TBX5) to induce cardiogenesis [99]. This method directly resulted in direct reprogramming of human dermal fibroblasts into cardiomyocytes, which were, in turn, injected into in vivo mouse models for regeneration. The conversion of normal human dermal fibroblasts into cardiomyocytes was confirmed histologically and by quantification of cardiac troponin I and α-actinin found in mature cardiomyocytes.

## 6. Magnetic Nanoparticles

Magnetic nanoparticles composed of iron oxide have briefly been investigated as well. These systems have been particularly helpful in manipulation of cells or targeting applications. In one such study, magnetic ferumoxytol nanoparticles were used to label cardiosphere-derived stem cells and to target them into coronary arteries of rat post-myocardial infarction ischemia/reperfusion models, thereby addressing the ordinarily low level of stem cell retention and engraftment [100]. In another study, magnetic iron oxide nanoparticles have also been utilized in guiding tissue scaffold structure following implantation into a rat model [101]. In this study, Zwi-Dantsis et al. demonstrated that magnetic iron oxide nanoparticles conjugated to antibodies directed at signal-regulatory protein alpha (SIRPA) could be utilized to manipulate human cardiomyocytes in situ with magnets. Chouhan et al. used iron oxide nanoparticles in another manner, building a magnetic actuator device using a silk fibroin matrix loaded with drug and iron oxide nanoparticles to stimulate differentiation of embryonic H9C2 myoblast cells into cardiomyocytes [102]. These studies uniquely harnessed the magnetic properties of iron oxide for cellular manipulation both in vitro and in vivo.

Iron oxide nanoparticles have been found to have beneficial properties on cardiomyocyte growth as well. Supraparamagnetic iron oxide nanoparticles (SPIONs) have been incorporated into silk-fibroin nanofibers to promote proliferation of mouse embryonic cardiac cells [103]. Like other nanoparticle systems, there was a noted upregulation of GATA-4, cardiac troponin T, Nkx 2.5, and α-MHC in embryonic cardiac cells grown on SPION-containing scaffolds versus those grown on silk fibroin without SPIONs. Iron oxide nanoparticles also stimulate connexin 43 expression in order to promote co-culture of mesenchymal stem cells and cardiomyoblasts, resulting in increased therapeutic efficacy [104]. Mou et al. had similar findings [105]. Thus, iron oxide nanoparticles do have a direct effect on cell signaling and phenotype.

## 7. Other Nanoparticles

Various other nanoparticles have also been used in cardiac tissue engineering. Silicon NPs modified with atrial natriuretic peptide (ANP) have been used to target cardiomyocytes both in vitro and in vivo [106]. In cell culture, ANP-modified silicon NPs were taken up at high rates by H9C2 myoblasts and primary cardiomyocytes relative to untargeted control NPs, especially in normoxic conditions. In a myocardial infarction rat model, NPs targeted with ANP or other targeting peptide sequences taken up at higher rates than untargeted NPs, illustrating that modification of nanoparticles could potentially be useful in targeting NPs to heart tissue.

Motivated by the presence of lower selenium concentrations in patients with cardiomyopathy, Kalishwaralal et al. designed chitosan-selenium nanoparticles for cardiac patches with enhanced electrical conductivity and mechanical strength [107]. This film was able to support growth and proliferation of H9C2 rat heart myoblasts in vitro.

Liu et al. developed another chitosan-based cardiac patch formulation composed of TiO2 NPs in PEG-chitosan hydrogels [108]. Young’s moduli increased and swelling ratios decreased with the addition of TiO2 NPs. NRVM cells grown on TiO2 NP-containing PEG-chitosan hydrogels exhibited similar viability and qualitatively improved synchronous activity in comparison to hydrogels without TiO2 NPs.

## 8. MicroRNA Delivery

**Figure 4:**
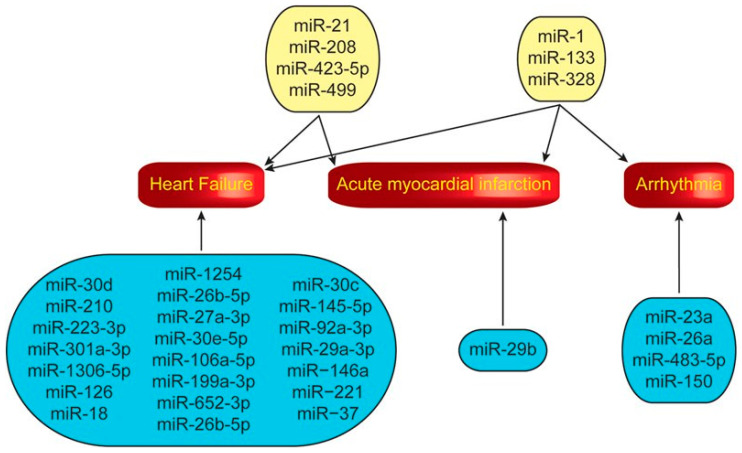
MicroRNAs (miRNAs) associated with heart failure, acute myocardial infarction, and arrhythmias. miRNAs in blue boxes correspond to those associated with only one of the three conditions while miRNAs in yellow boxes correspond to those associated with multiple conditions [110]. Copyright 2018 Springer Nature.

MicroRNAs (miRNAs) are single-stranded noncoding RNA strands, usually 20–25 nucleotides in length, which are capable of downregulating target messenger RNA (mRNA) through RNA interference [109,110]. Studies investigating the mechanism of action of miRNAs have shown that they play an important role in post-translational gene regulation, affecting a number of cellular processes including development, differentiation, proliferation, apoptosis, and tumorigenesis [111]. A number of studies have shown that miRNAs can function as oncogenes or tumor suppressors in certain conditions, and dysregulation of these miRNAs can affect proliferative signaling, invasion, and metastasis [112]. Additionally, there is increasing evidence that miRNAs can be used as diagnostic biomarkers for cardiovascular disease and may represent novel therapeutic targets for several cardiovascular disorders [110]. miRNAs are also thought to be involved in the development and progression of heart failure (Figure 4) [110]. Studies investigating the role of miRNAs in cardiac disease indicate that there is a role for the use of miRNAs as diagnostic biomarkers in heart failure, as well as potential therapeutic targets.

Multiple miRNAs have been shown to be important in cardiac disease and cardiac repair. A study utilizing high-throughput screening to identify miRNAs involved in cardiomyocyte proliferation in rats showed that miR-590 and miR-199a promote ex vivo cardiomyocyte proliferation. Following myocardial infarction in mice, administration of these miRNAs stimulated cardiac repair with near complete recovery of cardiac function [113]. A pig model was used to test the therapeutic effect of delivering miR-199a following ischemia and reperfusion, and it was shown to promote cardiomyocyte proliferation and myocardial repair with near complete recovery of cardiac functional parameters over three months [114]. As such, it represents a promising therapeutic candidate to promote cardiac regeneration following ischemic injury.

The main challenges of delivering miRNA therapy are that free RNA shows poor cellular uptake, short half-life, and significant off-target effects. These challenges make the delivery of free RNA for therapeutic purposes impractical. There have been many attempts to modify miRNAs to increase their stability and cellular uptake; however, off-target effects and the formation of toxic byproducts by degradation remain problematic. Alternative strategies such as the use of lipid formulations for the delivery of miRNAs has shown promise, but traditional transfection agents can have significant toxicity [115]. Nanoparticle-based carriers have been successfully used in a number of applications for the delivery of nucleic acids and show promise for use in cardiac applications. Studies have shown promise for the use of exosomes isolated from cardiac stem cells as a therapeutic agent to stimulate cardiac regeneration. These exosomes have been shown to contain miRNAs such as miR-146a, which is known to suppress post-myocardial-infarction injury by attenuating inflammation [116]. A second recent study showed potential for the use of a biomaterial that combines a shear-thinning hydrogel with DSPE-PEG (distearoylphosphatidylethanolamine- polyethylene glycol) nanoparticles conjugated to cardioprotective miR-199a-3p for use, following myocardial infarction [117]. This system allows for minimally invasive delivery of nanoparticles that can be targeted to the infarct site [118]. In an ischemia/reperfusion rat model, this system allowed a fast injection into the target site with localized retention of the miRNA-nanoparticle conjugates, eventually leading to improved cardiac function over a 3-month period [117]. Another recent study investigated the effect of delivering nanoparticles composed of hyaluronan-sulfate and miR-21 to cardiac macrophages on cardiac repair and remodeling. Treatment with these constructs following myocardial infarction in a mouse model showed increased angiogenesis and decreased hypertrophy and fibrosis, indicating that the miR-21 mimic acts to attenuate remodeling and may improve cardiac function long-term [119]. These studies indicate that there is significant promise for the use of nanoparticle-based miRNA delivery in treatment of myocardial infarction to promote cardiac regeneration and prevent post-ischemic heart failure.

## 9. Conclusions and Future Directions

There has been a tremendous recent effort in the development of nanoparticle-containing materials for cardiac tissue engineering, much of which now focuses on the translation of these materials toward clinical solutions. The nanoparticles that have been most studied to date are carbon nanotubes, gold nanoparticles, and gold nanorods. These materials have successfully directed greater proliferation of cardiomyocytes and enhanced cardiomyocyte function. More recently, there have been efforts to deliver bioactive compounds via nanoparticles within these scaffolds. Other innovative approaches including magnetic in vivo manipulation of cardiomyocytes using iron oxide nanoparticles have also been attempted. Even though clinical data regarding the use of these nanomaterials is still lacking, there has been a recent emphasis on translational research that has involved the use in vivo models. These promising translational studies motivate clinical evaluation of these materials in the near future.

As the nanomaterials community moves toward consideration of these materials in clinical research, there are several additional directions for future research in vitro and in vivo. First, greater mechanistic insights will be helpful in optimizing material design for cardiac regeneration. The addition of nanomaterials into bulk materials often change more than one property alone, including mechanical properties, electrical conductivity, characteristics of contact with cells, and roughness. However, relatively few efforts have been made to determine which properties are most relevant. Moreover, the biological mechanism by which cell phenotype is altered by nanoparticles alter cell phenotype must be further studied. Second, additional work will need to be done to assess the long-term safety and stability of these materials in vivo. Third, the delivery of biologically active molecules from nanoparticles for cardiac tissue engineering, such as miRNA, presents great promise to promote healing and merits deeper exploration. Building upon current nanoparticle research efforts in cardiac tissue engineering into these directions would be exciting for the treatment of ischemic heart disease and advanced heart failure, potentially representing an alternative to left ventricular assist devices and heart transplant.
